# NIPS: Network Inference with Partial State measurements using forced-delay embedding

**DOI:** 10.1093/pnasnexus/pgaf397

**Published:** 2025-12-24

**Authors:** Bharat Singhal, István Z Kiss, Jr-Shin Li

**Affiliations:** Department of Electrical & Systems Engineering, Washington University in St. Louis, St. Louis, MO 63130, USA; Department of Chemistry, Saint Louis University, St. Louis, MO 63103, USA; Department of Electrical & Systems Engineering, Washington University in St. Louis, St. Louis, MO 63130, USA

**Keywords:** network reconstruction, time-delay embedding, nonlinear networks

## Abstract

Decoding the connectivity patterns of complex networks from time series measurements is crucial for understanding and controlling their dynamics. Although network inference algorithms have advanced significantly in identifying both pairwise and higher-order interactions, they often rely on the availability of full-state measurements, an assumption that is difficult to satisfy in practice. In this article, we address this limitation by introducing Network Inference from Partial States (NIPS), a framework for network reconstruction from partial-state observations of network units. Focusing initially on networks coupled through observable states, we model coupling inputs as external forcing and utilize forced-delay embedding theory to establish a map that describes the evolution of the node observables as a function of observable state components. Specifically, the dynamics of the observable state of a node depends only on delayed observations of that node itself, not on delayed observations of other nodes. This enables accurate network reconstruction with limited data, which is demonstrated using both simulated and experimental data obtained from a wide range of networks. We evaluate the robustness of NIPS to noisy data and hidden network nodes and subsequently extend the framework to networks coupled through unobservable states.

Significance Statement Determining the effective network structure from time series measurements is a common theme across various scientific domains, from neuroscience and biology to chemistry. Existing network reconstruction methods typically require measurements of all variables describing each unit, such as both the membrane voltage and ion concentrations of a neuron, or the concentrations of all reactants in a chemical reaction. This limits their applicability to practical scenarios where only a few variables can be measured. The novel contribution of this work is the development of a network reconstruction approach that requires only one state measurement of each unit in the network, without prior knowledge of the node- or coupling-dynamics. We demonstrate that our method is capable of recovering network interactions using partial-state observations in various systems with different timescales, ranging from seconds to days.

## Introduction

Complex networks of high-dimensional nonlinear units are prevalent in natural and human-built environments, exhibiting a wide array of intriguing phenomena such as synchronization (1) and self-organized patterns (2). Understanding these rich behaviors and effectively controlling them by identifying optimal control sites requires decoding the underlying network connectivity from time series measurements of the nodes (or units). In circadian biology, for example, elucidating the cellular connectivity of the suprachiasmatic nucleus (SCN) is critical to uncovering the mechanisms driving its synchronization or splitting behavior (3–5), which has applications in jet-lag recovery (6). Similarly, in neuroscience, determining the wiring diagram of neuronal circuits is essential for understanding the information-encoding principles of the brain (7), detecting epileptic seizures (8), and developing potential treatments for neurological disorders (9).

Recent network inference algorithms can accurately determine the network structure from node state measurements (10–15); however, these algorithms require full-state node measurements. This is because the principal idea behind these system-theoretic approaches is to find the nonlinear equations governing the network evolution by expressing the unknown network dynamics as a linear combination of known basis functions. The network connectivity is then determined by finding the coefficients of these basis functions using linear regression and its variants with various penalty functions (e.g. $l_{1}$ (16) penalty). The primary distinction between different approaches arises from the assumptions on the network model considered. For example, in (13, 15, 17), network models with pairwise interactions are considered, and connectivity is determined using truncated singular value decomposition (SVD) (15) or alternating least squares (13). The assumption of pairwise coupling is relaxed in Ref. (11) by considering a general network model consisting of higher-order interactions, and the network structure is then inferred by using block orthogonal least squares. In Ref. (14), the network is assumed to have a scale-free structure (18) with chaotic dynamics, enabling the use of a mean-field approach for data-efficient network reconstruction. In Ref. (19), a two-step procedure is proposed that combines a correlation statistic with a model-fitting procedure to reduce the unknown variable count, thus improving the inference accuracy for oscillator networks. An ergodic basis pursuit approach is proposed in Ref. (12) for weakly coupled sparse networks with chaotic dynamics, where the minimum time series length for network reconstruction scales logarithmically with network size.

The assumption on full-state measurements presents a significant challenge in practice, as the nodes are high-dimensional systems, and only a subset of node states, often one, can be measured. Specifically, let $X_{i}\in R^{n}$ (n≥2) be the state of node *i*, then only a component of $X_{i}$, say $x_{i}\in R$, can be recorded. Numerous such examples exist across various fields such as electrochemistry, neuroscience, and engineering. For example, in electrochemistry, typically one variable, the electrode potential or the current, is measured, and other variables (oxide coverage or surface ion concentrations) are hidden (20). In AC power grid networks, frequency measurements are readily available, whereas determining phases of oscillatory units is challenging (21). In brain networks, EEG recording can only measure the electric field of neurons, which are multidimensional systems; for example, the Hodgkin–Huxley neuron model is a 4D system (22).

We note that information-theoretic methods, such as correlation (23), mutual information (24), maximal information coefficient (3), transfer entropy (25), a combination of mutual information and transfer entropy (26), and Granger causality (27), only require a single node state to reconstruct network connectivity. However, these methods recover functional connectivity (i.e. statistical dependencies), which may be different from the true or effective network structure (28, 29).

In this article, we introduce a framework for network reconstruction from scalar observations of high-dimensional node states by integrating time-delay embedding with statistical learning techniques. Many types of networks, such as electrochemical oscillators (30), circadian gene expression models (31), and neuronal networks (32), are coupled through observable node states. Our approach, Network Inference from Partial States (NIPS), assumes such a scientific setting without prior knowledge of the node dynamics or the coupling functions. We model the coupling inputs as external forcing, which enables us to leverage forced time-delay embedding theory. Consequently, it is sufficient to only embed the state of the specific node (for which we seek to infer connections) rather than embedding the entire network states, resulting in accurate network reconstruction with fewer data samples. We demonstrate the versatility of NIPS by reconstructing networks across various domains, including neuroscience (neuronal networks), circadian biology (SCN networks), chemistry (electrochemical oscillators), and engineering (electronic Rössler oscillators). Our findings show that NIPS reconstructs networks more accurately than commonly used functional inference methods, achieving performance comparable to that of a full-state measurement scenario. We further extend our approach to networks coupled via unobservable states.

## NIPS algorithm

A dynamic network of *N* nonlinear units with pairwise interactions can be modeled as


(1) $${\dot{X}}_{i}(t)=f_{i}(X_{i}(t))+\sum_{\begin{array}{c} \;j=1\\ j\neq i \end{array}}^{N}H_{ij}(X_{i}(t),X_{j}(t)),\;i=1,\ldots ,N,$$

where $X_{i}\in M\subset R^{n}$ is the state of node *i* evolving on a smooth compact manifold M, $f_{i}\;:\;M\to M$ is a nonlinear function describing the self-dynamics of node *i*, and $H_{ij}\;:\;M\times M\to M$ corresponds to coupling input from node *j* to node *i* if there is a connection; otherwise, $H_{ij}=0$. The directed network topology is represented by $H_{ij}$, where a nonzero $H_{ij}$ indicates the presence of a connection from node *j* to node *i*.

We assume that both drift and coupling functions $f_{i}$ and $H_{ij}$ are smooth functions that are unknown. In practice, it is often infeasible to measure the full state of each node ($X_{i}$, i=1,…,N), and only a single component of $X_{i}$, say $x_{i}$, is observable (see examples in the Introduction). Specifically, let $X_{i}=(x_{i,1},\ldots ,x_{i,n}{)}^{\prime }$, where ${\;}^{\prime }$ denotes the transpose; then only the *k*th component of $X_{i}$, i.e. $x_{i}=x_{i,k}$, can be recorded. Now, given the time series for each node $x_{i}(s\Delta t)$, where i=1,…,N, s=0,…,M+1, and Δt and M+2 denote the sampling interval and total samples, our goal is to efficiently infer network connectivity. Note that we assume all the network nodes are sampled simultaneously. In applications where data from different nodes are sampled at distinct times, a preprocessing step can be added to resample the data onto a shared time grid using interpolation techniques such as splines or Gaussian processes.

To begin, we first consider that the network Eq. 1 is coupled only through the observable states; we will address coupling via unobservable states later. Specifically, the state of the node *i* evolves according to


(2) $${\dot{X}}_{i}(t)=f_{i}(X_{i}(t))+\sum_{\begin{array}{c} \;j=1\\ j\neq i \end{array}}^{N}H_{ij}(X_{i}(t),x_{j}(t)),\;i=1,\ldots ,N,$$

where the coupling function $H_{ij}(\cdot )$ only depends on the observable state of node *j*. This assumption is inspired by a variety of nonlinear models that describe networks across diverse domains such as chemistry, neuroscience, and circadian biology. Examples include Hodgkin–Huxley neural networks with synaptic or gap junction coupling, where the observable state is the action potential (32); electrochemical oscillators, where the electrode potential is the measurable state (30); electrically coupled Rulkov maps that model bursting neural dynamics with coupling through the action potential (33); and transcription-based oscillators that describe circadian gene expression, where coupling influences the mRNA transcription rate of a cell (31).

We address the partial measurement bottleneck by leveraging time-delay embedding, which reconstructs unknown dynamical systems from scalar time series observations by forming a different state space with delayed measurements (34, 35). However, the high dimensionality of the network presents challenges to the direct application of this approach. We first discuss these challenges and then describe our approach.

### Delay embedding for autonomous systems

Consider a *n*-dimensional state vector x evolving according to a deterministic dynamic law $\dot{x}(t)=f(x(t))$ on a compact *n*-dimensional manifold. When the vector field f(⋅) is unknown and only a scalar measurement y(t) can be recorded according to an unknown observation function φ(x(t)), i.e. y(t)=φ(x(t)), one common task is to reconstruct a copy of the original system. This is achieved by considering delayed observations of y(t), given by $y_{d}(t)=[y(t),y(t-\tau ),\ldots ,y(t-(k-1)\tau ){]}^{\prime }$ as a proxy of the state x(t), and recovering the equations describing the evolution of $y_{d}(t)$, i.e. ${\dot{y}}_{d}(t)=F(y_{d}(t))$.

This follows from Takens’ theorem (35), which, informally, states that for generic *f* and φ, and k≥2n+1, the dynamical system described by *F* is equivalent to $\dot{x}(t)=f(x(t))$ up to a smooth invertible coordinate change; hence, all coordinate-independent properties of *f* and *F* will be identical (36). The significance of the Takens’s theorem is that while the original system may be unobservable, many properties of *f* can still be inferred solely from samples of y(t) (by reconstructing *F*). However, for its validity, both *f* and φ must be autonomous, i.e. independent of any outside influence.

Remark 1 We note that theoretically, any delay (*τ*) choice will result in a valid embedding as long as the embedding dimension *k* is large enough and the measurements are noiseless; in practice, however, different choices of *τ* and *k* can produce varying results (i.e. reconstruction quality of *F*) (37). Several methods exist for the evaluation of appropriate embedding parameters (*τ* and *k*); for example, false nearest neighbors (38), method of characteristic lengths (39), average mutual information (40), and wavering product (41). Nonuniform delays can also be used to define the delayed state variable $y_{d}(t)$, i.e. $y_{d}(t)=[y(t-\tau_{1}),y(t-\tau_{2}),\ldots ,y(t-\tau_{k}){]}^{\prime }$ (Ref. (42)).

### Embedding for network dynamics

Let $X=(X_{1},\ldots ,X_{N}{)}^{\prime }\in M^{N}\subset R^{N\times n}$ denote the state of the network, evolving according to $\dot{X}=F(X)$, where the *i*th component of F is given by Eq. 2. We observe the network through the observation function $\varphi (X)=(x_{1},\ldots ,x_{N}{)}^{\prime }\in R^{N}$.

As the observation function is multidimensional, an embedding dimension k≥(2n+1) suffices to reconstruct the dynamics as Nk>2Nn (34, 43). Consequently, the dynamics of observable state in terms of delayed observations is given by


(3) $$\begin{array}{rl} {\dot{x}}_{i}(t) & =F_{i}(x_{1}(t),x_{1}(t-\tau ),\ldots ,x_{1}(t-(k-1)\tau ),\\ & \;x_{2}(t),x_{2}(t-\tau ),\ldots ,x_{2}(t-(k-1)\tau ),\\ & \;\vdots \\ & \;x_{N}(t),x_{N}(t-\tau ),\ldots ,x_{N}(t-(k-1)\tau )). \end{array}$$

Network connectivity can then be determined by estimating $F_{i}$ from the measurement data; however, estimation of $F_{i}$ requires considering delayed observations of all observable states of the nodes, necessitating a large quantity of data (long time series) due to the curse of dimensionality (see Ref. (44) for the scaling relationship between time series length and number of delayed observations for nonparametric estimation). In practice, obtaining such long time series is often infeasible. For example, in electrochemical oscillator networks, frequency drift limits the time over which data can be recorded (45); in neuronal networks, the length of in vitro recording data is restricted by tissue degradation (26).

### Delay embedding for forced systems with known forcing

As noted, Takens’ theorem applies to autonomous systems, i.e. when both *f* and φ are independent of any external influence. In many practical scenarios, however, the evolution of the state vector x∈M is influenced by an external deterministic forcing u(t), independent of x(t), and knowledge of the forcing is available. In other words, $\dot{x}(t)=f(x(t),u(t))$ and the forcing u(t) is known. Consequently, it suffices to embed each M×{u(t)}, since the forcing dynamics do not need to be reconstructed. Under these conditions, the evolution of a scalar measurement y(t)=φ(x(t)) can be expressed as $\dot{y}(t)=G(y(t),y(t-\tau ),\ldots ,y(t-(k-1)\tau ),u(t))$, where k≥2n+1, as shown in Stark et al. (36). The key advantage of this formulation is that, since u(t) is known, one does not need to reconstruct the input dynamics, thereby reducing data requirements.

### Forced-delay embedding for decoding network connectivity

Instead of embedding the network directly, we exploit the fact that the coupling input depends only on the observable states of each node, as represented in Eq. 2. Rather than embedding the entire network, we propose to embed each node individually. Note that Takens’s theorem cannot be applied, as it requires the system to be autonomous; however, each node’s state $X_{i}$ is influenced by other node states, introducing nonautonomous behavior. To embed each node separately, we approximate the dynamics of each node as a forced system, treating the coupling inputs from the remaining N−1 nodes as an external forcing coming from a deterministic system; namely, we approximate Eq. 2 as


(4) $$\begin{array}{cc} {\dot{X}}_{i}(t) & =f_{i}(X_{i}(t))+H_{i}(X_{o}^{\left(\bar{i}\right)}(t),X_{i}(t)),\\ {\dot{X}}_{o}^{\left(\bar{i}\right)}(t) & \approx Z_{i}(X_{o}^{\left(\bar{i}\right)}(t),X_{no}^{\left(\bar{i}\right)}(t)), \end{array}$$

where $X_{o}^{\left(\bar{i}\right)}(t)=[x_{1}(t),\ldots ,x_{\;j\neq i},\ldots ,x_{N}(t){]}^{\prime }\in R^{N-1}$ and $X_{no}^{\left(\bar{i}\right)}(t)\in R^{\left(n-1)\times (N-1\right)}$ contains observable and nonobservable states of all the nodes except *i*, and $H_{i}(X_{o}^{\left(\bar{i}\right)}(t),X_{i}(t))=\sum_{\;j=1}^{N}H_{ij}(X_{i}(t),x_{j}(t))$. The evolution of forcing $X_{o}^{\left(\bar{i}\right)}(t)$ is described by some $Z_{i}(\cdot )$, which we assume to be not dependent on $x_{i}(t)$. Equation 4 approximates Eq. 2, with the only difference being the assumption that the evolution of the forcing term is independent of $x_{i}(t)$. Under this approximation, the network is conceptualized as a coupled system, wherein node *i* forms one subsystem and the remaining N−1 nodes constitute the other. The influence of node *i* on the second subsystem is considered negligible; that is, the removal of all outgoing connections from node *i* is assumed to have only a marginal impact on the overall network dynamics. This formulation enables the dynamics of node *i* to be regarded as those of a forced system, with the forcing treated as independent of the node *i* state.

By expressing the dynamics of the node *i* as a forced system (Eq. 4) with known forcing $\left(X_{o}^{\left(\bar{i}\right)}\right)$ having deterministic dynamics, we can utilize the theory of delay embedding for forced systems (36, 46). Specifically, we embed each $M\times \{X_{o}^{\left(\bar{i}\right)}(t)\}$ as independent measurements of the forcing $X_{o}^{\left(\bar{i}\right)}(t)$ are available. As a result, the equivalent map describing the evolution of $\varphi (X_{i})=x_{i}(t)$ can be represented in the delayed coordinates of $x_{i}(t)$ alone, as


(5) $${\dot{x}}_{i}(t)=F_{i}(x_{i}(t),x_{i}(t-\tau_{i}),\ldots ,x_{i}(t-(k-1)\tau_{i}),X_{o}^{\left(\bar{i}\right)}(t)),$$

where the number of delays k≥2n+1 and $\tau_{i}$ is determined from the sampled data $x_{i}(s\Delta t)$. The main advantage of this approach is that we only need to consider the delayed observations of $x_{i}$, instead of all the network states (Eq. 3). This could be significantly advantageous for large networks.

Next, to infer the connectivity of the network, we determine the functions $F_{i}$, i=1,…,N. To this end, we first decompose $F_{i}$ into additive form, i.e.


(6) $${\dot{x}}_{i}(t)=G_{i}(x_{i,d}(t))+\sum_{\begin{array}{c} \;j=1,j\neq i \end{array}}^{N}g_{ij}(x_{j}(t)),$$

where $x_{i,d}(t)=(x_{i}(t),x_{i}(t-\tau_{i}),\ldots ,x_{i}(t-(k-1)\tau_{i}){)}^{\prime }$. Such additive assumptions are common for high-dimensional nonparametric regression (see Refs. 47, 48) and are advantageous even when there is interaction between the variables due to reduced model variance (49). The nonlinear functions $G_{i}$ and $g_{ij}$ are estimated by first expressing them as a linear combination of known basis functions and then obtaining the basis function coefficients by fitting the measurement data to Eq. 6 (see Methods). We obtain a weighted matrix after fitting the data to Eq. 6, where the (i,j)th element of the matrix ${\hat{k}}_{ij}$ is the weight of the connection from node *j* to node *i*. Consider a 100 node network, with each node being a 3D system (n=3). Using a third-order polynomial basis, the number of unknown coefficients is reduced from 11,901 to 417 by embedding only the individual nodes instead of the entire network. This reduction occurs because the number of unknown coefficients decreases from N(k+rk)−(N−1) to (k+rk)+(N−1)r, where *r* is the polynomial order and k(=2n+1) is the number of delays (see Methods).

We now demonstrate the applicability of NIPS to networks originating from diverse domains, characterized by dynamics ranging from periodic to chaotic with time scales from seconds to days. These include electrochemical oscillators, neuronal networks, circadian clock networks, and electronic Rössler networks.

## Inference of electrochemical oscillators

We consider directed networks of 100 electrochemical oscillators with four incoming connections per node. These oscillators describe the nickel electrodissolution reaction in sulfuric acid and are modeled as (20)


(7) $$\begin{array}{rl} \frac{dV_{i}}{dt} & =\frac{U-V_{i}}{R}-\left[\frac{C_{h}e^{0.5V_{i}}}{1+C_{h}e^{V_{i}}}+ae^{V_{i}}\right]{\bar{\nu }}_{i}+\sum_{\;j=1}^{100}k_{ij}a_{ij}\Delta V_{ij},\\ \frac{d\nu_{i}}{dt} & =\frac{e^{0.5V_{i}}}{\Gamma (1+C_{h}e^{V_{i}})}{\bar{\nu }}_{i}-\frac{bC_{h}e^{2V_{i}}}{\Gamma (cC_{h}+e^{V_{i}})}\nu_{i}, \end{array}$$

where $\Delta V_{ij}=V_{j}-V_{i}$, ${\bar{\nu }}_{i}=1-\nu_{i}$, and $V_{i}$ and $\nu_{i}$ correspond to the electrode potential and the surface coverage of the passivating oxide species of the oscillator *i*. The binary variables $a_{ij}$ indicate the presence or absence of a connection and $k_{ij}\in R^{+}$ denotes the strength of the connection from node *j* to node *i*. The model parameters *U*, *R*, $C_{h}$, *a*, *Γ*, *b*, *c*, and the coupling strengths $k_{ij}$ are provided in Supplementary Table S1.

**Fig. 1.**
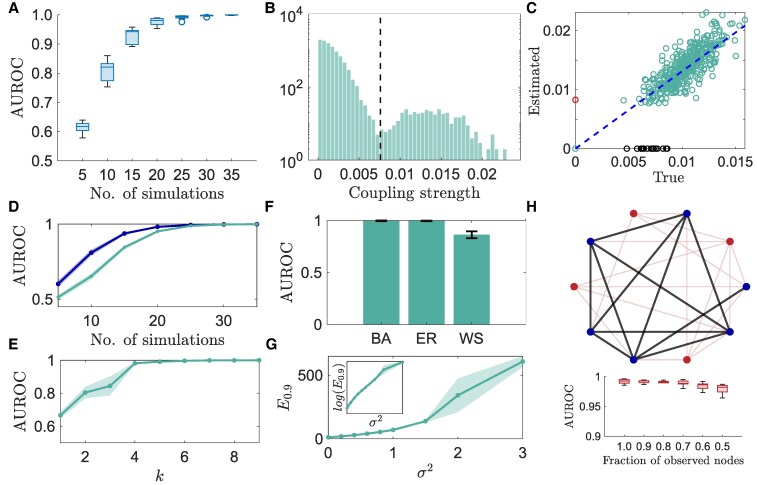
Inference of electrochemical oscillator networks and performance analysis of the NIPS algorithm. A) AUROC score as a function of measurement data length, collected from independent simulations. B) Histogram of inferred coupling strengths for a representative network, with the dotted black line indicating the threshold used to determine network connectivity. C) Positive correlation $\left(r^{2}\in [0.95,0.99\right]$ between true and estimated coupling strengths, where red and black circles represent false positives and false negatives, respectively. D) Comparison of AUROC scores with full (blue) and partial (green) state measurement scenarios. Algorithm ICON (15) is used to reconstruct the network using the complete state measurements. E, F) Impact of embedding dimension *k* and the network topology on reconstruction performance, respectively. G) Number of simulations required to achieve AUROC ≥0.9 ($E_{0.9}$) as measurement noise intensity (evaluated by $\sigma^{2}$) increases, with the inset showing $E_{0.9}$ on a log scale. H) Sample network where observed nodes are colored blue and hidden nodes red; only links between observed nodes (black lines) require recovery. The plot shows AUROC variation as different fractions of nodes are observed. The sample size is 10, with each network having 100 nodes, and the algorithm hyper-parameters are kept identical.

We generate data by simulating the model equations 35 times, where each simulation starts from random initial conditions, and data ($V_{i}$) is recorded for 5 cycles with 100 samples per cycle. Multiple simulations are conducted to generate information-rich datasets. The network is reconstructed using NIPS by taking the embedding dimension k=5 as each oscillator is a 2D system, and the embedding delay $\tau_{i}$ is determined from $V_{i}(t)$ using the average mutual information algorithm (50). The network reconstruction accuracy (measured using the area under the receiver operating characteristic curve (AUROC) score; see Methods) for data obtained from multiple simulations is shown in Fig. 1A. We find that our method can recover network connections accurately with only measurements of electrode potentials. The inference accuracy improves as we use data from a larger number of simulations. The benefit of embedding the node states individually rather than embedding the entire network (as in Eq. 3) is demonstrated by the difference in sample points required to achieve an AUROC ≥0.9: 7,500 versus 17,500 (Supplementary Table 2). This disparity becomes more pronounced as the network size is increased to 500 nodes, requiring 15,000 sample points compared to 37,500. We further examine the relationship between the inferred and true coupling strengths. The histogram of the recovered coupling strengths, ${\hat{k}}_{ij}$, is presented in Fig. 1B, where the black dotted line indicates the optimal threshold, determined using Otsu’s method, to identify network connectivity. Our analysis reveals a strong positive correlation between the inferred coupling weights and the true coupling weights, with a squared Pearson’s correlation coefficient of 0.98 (Fig. 1C). This suggests that the inferred coupling strengths closely reflect the true underlying interaction strengths.

NIPS can still recover the interactions, even when the measurements are a function of the observable state, i.e. we observe $\varphi (x_{i})$. We consider two different choices of φ(⋅): $\varphi (x_{i})=x_{i}^{2}$ and $\varphi (x_{i})=\frac{1}{1+e^{-x_{i}}}$. The number of samples to achieve AUROC ≥0.9 is 15,000 and 12,500, respectively (Supplementary Table S3). We also find that NIPS accurately reconstructs oscillator networks across various synchronization regimes, i.e. coupling strengths (Supplementary Fig. S3).

### Reconstruction performance

We characterize the performance of NIPS by considering different network topologies, measurement noise intensities, the number of embedding dimensions (k), and hidden units. We present four main categories of results. First, inference accuracy remains robust across three commonly observed network topologies: Erdős–Rényi (ER) random (51), Watt–Strogatz (WS) small-world (52), and Barabási–Albert (BA) scale-free (53) (Fig. 1F). This highlights that our approach is independent of the underlying network configuration. Second, reconstruction remains feasible for varying noise intensities, although the number of simulations (data points) required to achieve an AUROC score greater than 0.9 ($E_{0.9}$) grows exponentially (Fig. 1G). This observation is consistent with network inference methods that require full-state measurements (11, 13, 14). White Gaussian noise is added to the measurements $V_{i}(t)$. Third, optimal inference performance is achieved when k≥5, consistent with theoretical expectations, as k=5 is the minimum embedding dimension for a 2D system (Fig. 1E). Finally, even when some of the network nodes are hidden (i.e. $V_{i}(t)$ cannot be measured for certain *i*), the links between the observable nodes can still be reliably inferred (Fig. 1H). We observe a slight decrease in the AUROC score, estimated for the links between measured units, as the fraction of hidden units increases. This is because the hidden nodes have unknown coupling inputs to the measured units. Nevertheless, the reconstruction accuracy can be improved by increasing the length of the time series.

The computational cost for a 100 node sparse network (400 total connections) remains low with an inference time of 6.5±1s on a computer with an Intel (R) Core (TM) i7-10700 processor and 32 GB memory. The computational time can be further reduced by using parallel processing to infer the connectivity of each node simultaneously. This demonstrates the scalability and computational efficiency of NIPS for large networks.

### Effect of partial-state measurements

Next, we analyze the impact of missing state measurement by comparing the performance of NIPS to the algorithm “Inferring Connections of Networks (ICON) (15),” which requires full state observations. The results are shown in Fig. 1D for different lengths of time series (generated using independent simulations), where the blue (green) line corresponds to ICON (NIPS). For longer time series, the AUROC score is comparable to the full-state observation scenario. However, for shorter time series, a slight decrease in the AUROC score (up to 20%) is observed.

We also consider Rössler oscillator networks, where each node has chaotic dynamics and is described by a 3D system (54). We record a single state variable per node and analyze the reconstruction performance of our method. The results are similar to those of the electrochemical oscillator networks and are shown in Supplementary materials Section S2.

## Mouse visual cortex reconstruction

**Fig. 2.**
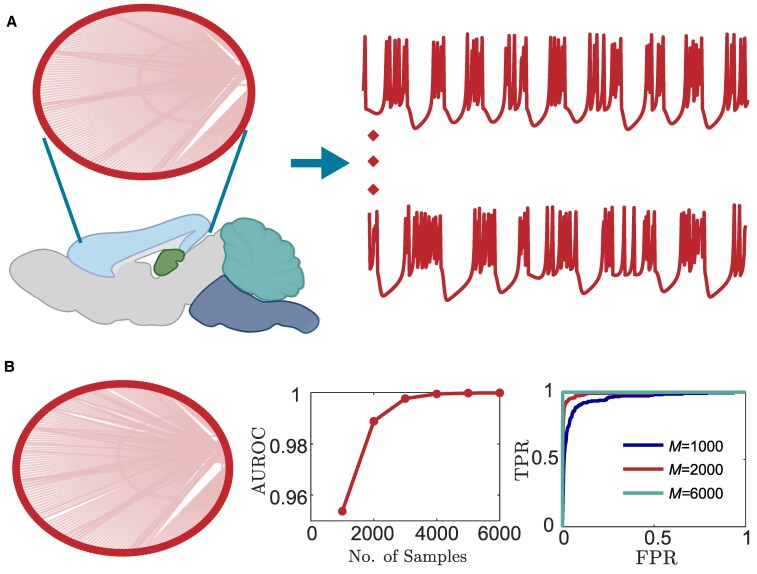
Reconstruction of a real mouse visual cortex network with neural dynamics modeled using Rulkov maps. A) shows network connectivity of the visual cortex brain region (left) and the recorded time series data (right) for two neurons (neuron 1 and 195). B) shows the reconstructed network, reconstruction accuracy with the length of sampled data, and the ROC curves for three different data lengths (from left to right). The AUROC scores corresponding to the blue, red, and green lines are 0.95, 0.98, and 1.0, respectively.

We analyze a directed neuronal network with 195 nodes and 214 edges, representing the connectivity of the visual cortex (55). The network dynamics are modeled using a coupled Rulkov map with diffusive coupling (14, 33) as


$$\begin{array}{rl} u_{i}(t+1) & =\frac{\beta }{1+u_{i}{\left(t\right)}^{2}}+v_{i}(t)+\sum_{\;j=1}^{195}k_{ij}a_{ij}(u_{j}-u_{i}),\\ v_{i}(t+1) & =v_{i}(t)-\nu u_{i}(t)-\sigma , \end{array}$$

where $u_{i}$ and $v_{i}$ denote the membrane potential (observable state) and the recovery variable (unobservable state) of the *i*th neuron, respectively. The binary variables $a_{ij}$ encode the network connectivity (Fig. 2A, left panel) and $k_{ij}\in [0.02,0.04]$ (uniformly distributed) denotes the strength of the connection. The model parameters β=4.2 and ν=σ=0.01 are constants to ensure spiking–bursting neural activity (33) (Fig. 2A, right panel).

To generate in silico data, we simulate the network six times from random (independent) initial conditions, where each simulation contains 1,000 samples of the membrane potential $u_{i}$ for each neuron. The network reconstruction accuracy for various data lengths, from 1,000 to 6,000, is shown in the middle panel of Fig. 2B. Different lengths of time series are obtained by combining data from multiple independent simulations. We find that even with only 1,000 samples and partial-state measurements, the reconstruction accuracy reaches 0.95 (where 0.5 corresponds to random guessing), while it requires 4-fold data length to achieve a similar accuracy by embedding the whole network. The receiver operating characteristic (ROC) curves for M=1,000,2,000, and 6,000 are shown in the rightmost panel of Fig. 2B. We note that NIPS outputs an estimated coupling matrix ${\hat{k}}_{ij}$, which is subsequently thresholded into a binary matrix using Otsu’s method (56). This thresholding step is performed to determine the reconstructed network, which is shown in the leftmost panel of Fig. 2B (M=6,000).

We further apply our method to networks of periodically spiking neurons, the FitzHugh-Nagumo neuron model (57). We observe that NIPS not only effectively identifies the underlying connectivity structure but can also accurately predict network dynamics (Supplementary material, Section S1).

## Inference of circadian clock networks

In mammals, circadian rhythms are governed by a network of ∼20,000 neurons in the hypothalamic SCN (5). Intercellular communication among these neurons synchronizes their rhythmic activity, thereby producing high-amplitude circadian gene expression that drives physiological rhythms (58, 59). Deciphering how these neuronal clocks achieve synchronization requires an understanding of the structure of cell-to-cell connectivity. Here, we demonstrate the effectiveness of NIPS in identifying directed intercellular connections in SCN networks.

We simulate a large network (300 cells), where cell *i* is modeled by a 3D differential equation, describing evolution of PERIOD2 gene mRNA $\left(M_{i}\right)$, cytosolic $\left(P_{C,i}\right)$, and nuclear PER2 protein $\left(P_{N,i}\right)$, given by


$$\begin{array}{rl} & {\dot{M}}_{i}(t)=v_{s,i}\frac{K_{1}^{n}}{K_{1}^{n}+P_{n,i}^{n}}-v_{m,i}\frac{M_{i}}{M_{i}+K_{m}},\\ & {\dot{P}}_{c,i}(t)=k_{s}M_{i}-v_{d}\frac{P_{c,i}}{k_{d}+P_{c,i}}-k_{1}P_{c,i}+k_{2}P_{n,i},\\ & {\dot{P}}_{n,i}(t)=k_{1}P_{c,i}-k_{2}P_{n,i}. \end{array}$$

The intercellular coupling is defined by the parameter $v_{s,i}=0.73+\sum_{\;j=1}^{300}a_{ij}k_{ij}(M_{j}-M_{i})$, where the coupling strengths $k_{ij}$ are optimized to ensure cells synchronize in 4–5 days, in line with experimental observations (3, 15). The number of cells and cellular heterogeneity in the period, induced by varying $v_{m,i}$, closely resemble those found in experimental SCN recordings (3, 15, 60). The remaining model parameters are identical for all cells (Supplementary Table 4), similar to Ref. (26).

**Fig. 3.**
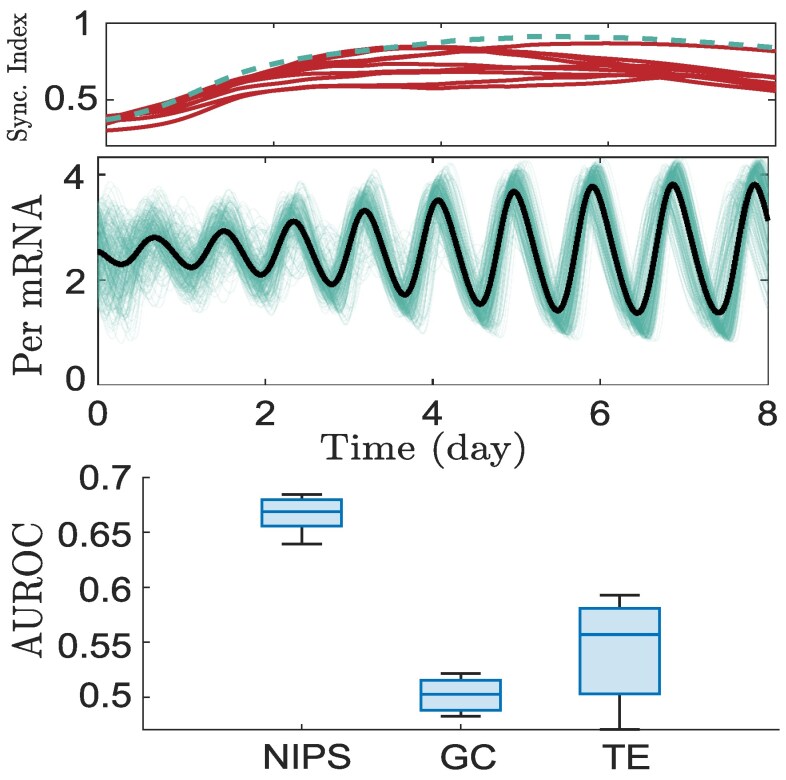
Reconstruction of the SCN network from in silico circadian gene expression data. The top panel shows the synchronization (sync.) index, estimated by the Kuramoto order parameter, for 10 analyzed networks. The middle panel displays the PERIOD2 gene mRNA for 300 cells (black line = mean) for a representative network, with the sync index shown by the dotted green line in the top panel. The bottom panel compares the inference accuracy of NIPS with GC and TE for all 10 networks. The inside line (similarly, the top and bottom edges) of the box plot represents the sample median (similarly, the upper and lower quartiles), while the top and bottom whiskers denote the maximum and minimum.

We generate 10 sample directed networks having small-world topology with hub nodes (i.e. nodes with high out-degree), consistent with findings that the SCN network exhibits both small-world characteristics (3) with hub structures (15). Each network is simulated for 8 days, and the expression of PERIOD2 gene mRNA is recorded with cells starting in a desynchronized state on day 0 and achieving synchronization in 4 to 5 days (Fig. 3). The sampling rate is 20 minutes per sample.

We infer directed connectivity from the recorded expression of the PERIOD2 mRNA gene $\left(M_{i}(t)\right)$ using NIPS and compare its performance with two widely used directed network inference methods in neuroscience: transfer entropy (TE) and Granger causality (GC) (25, 27). It is important to note that both TE and GC are designed to infer functional connectivity rather than effective connectivity (28). We estimate pairwise TE between all node pairs using the Python library “PyInform” and calculate GC using the Multivariate Granger Causality (MVGC) toolbox in MATLAB. Pairwise TE, as opposed to multivariate TE, is chosen for its reduced computational cost and improved accuracy for short time series (61). We find that NIPS outperforms the best-performing method between TE and GC by up to 30% (Fig. 3). This highlights the potential of applying NIPS to circadian gene expression data to infer directed network interactions, expanding the scope and accuracy of existing methods that focus on inferring undirected SCN connections (3).

## Validation on experimental data

We recover network interactions from experimental data recorded from undirected networks of 3D Rössler electronic oscillators (network size = 28), with only one observed state (62). Data were collected for various coupling strengths (that is, $k_{ij}$) between nodes across 20 different network topologies, with three separate time series acquired for each combination of coupling strength and network configuration. This resulted in a total of 2,000 combinations, comprising 100 coupling strengths for 20 network topologies (62). As the networks are undirected, we consider partial correlation (Pcor) and mutual information (MI), together with TE and GC, to compare the reconstruction performance. Only 20 combinations of coupling strengths are analyzed, as stronger coupling induced faster network synchronization, generating uninformative data (17).

**Fig. 4.**
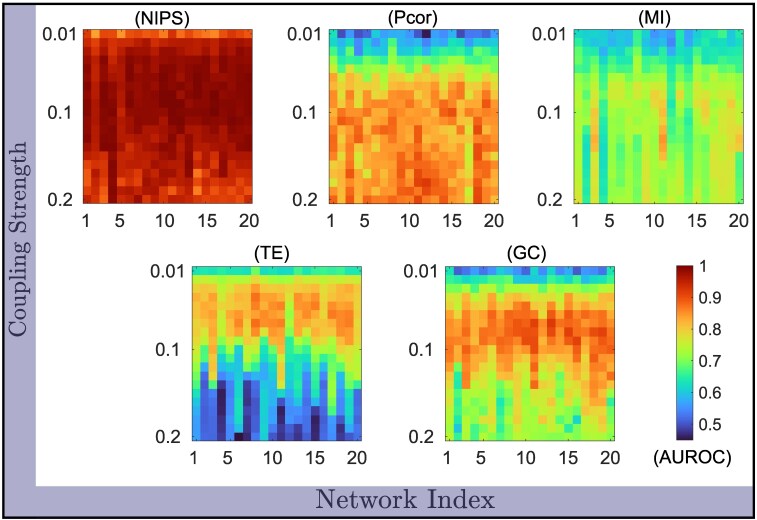
Accurate inference across various network structures and coupling strengths with noisy experimental data. The proposed method achieves better inference accuracy than commonly used network inference methods, including both directed and undirected approaches: partial correlation (Pcor), mutual information (MI), transfer entropy (TE), and Granger causality (GC), across combinations of 20 different network topologies and 20 different coupling strengths.

We find that NIPS consistently outperforms all four methods in all 400 network combinations, achieving nearly perfect reconstruction accuracy with an AUROC of 0.96±0.03 (mean ± SD, Fig. 4). In contrast, the AUROCs achieved by Pcor, MI, TE, and GC across all networks and coupling values were 0.78±0.1, 0.70±0.06, 0.68±0.12, and 0.77±0.09, respectively. Furthermore, NIPS demonstrates consistent performance across different network topologies and coupling values, as evidenced by its low SD, while the other approaches performed well only for specific combinations of coupling and network structures, as indicated by higher SD. The high AUROC observed for Rössler oscillators is for small networks (28 nodes) with small heterogeneity in the node dynamics; nevertheless, our approach is generalizable under large heterogeneity in node dynamics and network size (Supplementary material Sections 5.2 and 5.4).

## Coupling through unobserved states

**Fig. 5.**
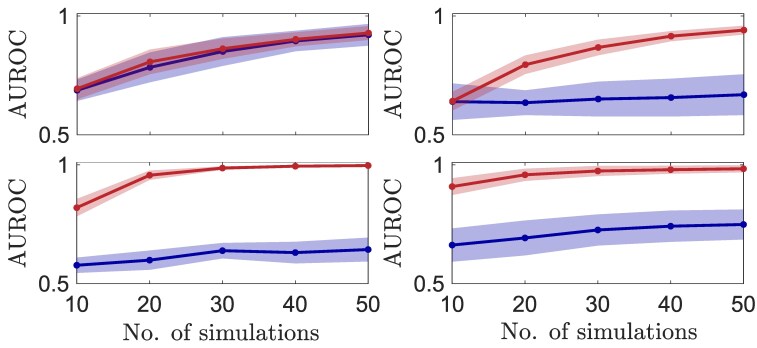
Network inference when nodes are coupled via unobservable states. The top-left, top-right, bottom-left, and bottom-right panels correspond to the coupling functions described by (a)–(d) in Eq. 8, respectively. The red (blue) line represents the mean AUROC for network (individual node) embedding. Sample size: 10; the shaded region denotes the SD.

Up to this point, we have recovered networks in which coupling inputs depend solely on observable states by embedding only individual node states. However, when the coupling involves unobservable states, embedding the individual node states is insufficient, and it becomes necessary to embed the states of the entire network using Eq. 3 for an accurate network reconstruction. We demonstrate this approach by examining a directed network of 30 electrochemical oscillators (Eq. 7) with four distinct coupling functions:


(8) $$\begin{array}{rl} & (a)H(X_{i},X_{j})=\left[\begin{array}{c} 0\\ a_{ij}k_{ij}\Delta \nu_{ij} \end{array}\right]\;(b)H(X_{i},X_{j})=\left[\begin{array}{c} a_{ij}k_{ij}\Delta \nu_{ij}\\ 0 \end{array}\right]\\ & (c)H(X_{i},X_{j})=\left[\begin{array}{c} a_{ij}k_{ij}\Delta V_{ij}\\ a_{ij}k_{ij}\Delta \nu_{ij} \end{array}\right]\;(d)H(X_{i},X_{j})=\left[\begin{array}{c} a_{ij}k_{ij}\Delta \nu_{ij}\\ a_{ij}k_{ij}\Delta V_{ij}, \end{array}\right] \end{array}$$

where $\Delta \nu_{ij}=\nu_{j}-\nu_{i}$ and $X_{i}=(V_{i},\nu_{i}{)}^{\prime }$ is the state of node *i*. We generate time series data by simulating electrochemical model equations and recover interactions by determining $F_{i}$ (embedding each node separately, Eq. 5) and $F_{i}$ (embedding the whole network, Eq. 3). The procedure for reconstructing network connections via estimation of $F_{i}$ from measurement data is described in Supplementary materials Section S3. The results are shown in Fig. 5, where the coupling inputs (a), (b), (c), and (d) correspond to the top-left, top-right, bottom-left, and bottom-right panels, and the red (blue) line represents the AUROC for network (individual node) embedding. These results demonstrate that accurate network reconstruction remains feasible even when nodes are coupled through unobservable states; in such cases, embedding the entire network is more data-efficient than embedding individual nodes, in contrast to the observable coupling case.

## Discussion

In this work, we present a framework for network reconstruction from scalar measurements of the node states. We primarily focus on networks where the coupling dynamics depends only on the observable node states. Rather than embedding the entire network dynamics, we embed the dynamics of each node independently, utilizing the theory of forced-delay embedding. This strategy allows the evolution of the observable state of node *i* to be described using only the delayed observations of node *i*, instead of the delayed observations of all network states, resulting in data-efficient inference. When the coupling involves unobservable states, we show that network connectivity can still be reconstructed by embedding the entire network. We demonstrate that our approach can reconstruct the network connectivity of networks with periodic and chaotic dynamics with both simulated and noisy experimental data, achieving performance comparable to full-state network inference methods and outperforming commonly used functional inference techniques.

In our analysis, we choose the embedding dimension as 2n+1, which requires knowledge of the system dimension. In practice, one may not know *n*; nonetheless, the embedding dimension could also be estimated from time series using, for example, the false nearest-neighbor algorithm (38). Our analysis (Fig. 1E) shows that NIPS is robust to the overestimation of *n*. Therefore, selecting a larger value of *n* serves as a practical strategy to account for estimation uncertainties. This makes our approach entirely data-driven, eliminating the need for any prior knowledge of the system dimension, node dynamics, or coupling functions, which could be particularly advantageous in practical scenarios. Our approach is differs from Ref. (21), which addresses the partial measurement problem for a specific class of 2D systems with known coupling functions.

From a computational standpoint, the NIPS approach is scalable to large networks, as connectivity inference for each node is performed independently and can be readily parallelized. For a given node, the primary computational cost arises from two steps: (i) estimating the appropriate embedding delay and (ii) solving a linear inverse problem (Eq. 10) to infer network connectivity. The embedding delay is estimated using the average mutual information (AMI) method, which has a computational complexity of $O(M\tau_{\max})$, where *M* is the number of samples and $\tau_{\max}$ is the maximum number of delays considered (63). Once the embedding is constructed, the least-squares problem involves solving an overdetermined linear system of size M×((k+rk)+(N−1)r1) (see Methods). This step can be performed efficiently using standard numerical linear algebra routines, with a computational complexity of $O(M^{2}N_{\mathrm{delayed}}+N_{\mathrm{delayed}}^{3})$, where Ndelayed=(k+rk)+(N−1)r1. Since $\tau_{\max}\ll M$ and *M* is larger than $N_{\mathrm{delayed}}$, the total computational complexity of NIPS can be obtained as O(M2(k+rk)+M2(N−1)r1). Furthermore, to ensure a unique solution to the linear system, the number of samples must satisfy M>(k+rk)+(N−1)r1. This implies that the number of samples required for accurate inference scales polynomially with the node dimension *n*, specifically as $O(k^{r}),k=2n+1$, and linearly with the network size *N*, provided the collected samples are sufficiently informative (i.e. not highly correlated) (64). This scaling behavior is also corroborated by our numerical experiments, which demonstrate a linear scaling of *M* with respect to *N* (Supplementary Fig. S6).

### Moving beyond reduced order models

For networks of periodic units, existing methods (Refs. (13, 15, 19, 65)) recover an equivalent reduced-order phase-model (66) that describes the evolution of phase obtained from observable node states. The network connections are then obtained from the phase connectivity. However, this approach has three main limitations: (i) reduced-order models are typically valid only for weakly coupled oscillators; (ii) phase estimation from time series is a challenge in itself, with errors in phase estimation translating directly into errors in network reconstruction; and (iii) information loss during phase transformation; for instance, phase estimation via peak-finding (67) retains only the phase at waveform peaks, omitting intermediate information. This is also evident in our analysis; when reconstructing the equivalent phase-model from experimental data (62), the mean AUROC score using the state-of-the-art inference method (Ref. (13)) is 0.87 for weak coupling (0.01 to 0.1), while our approach achieves 0.96. Moreover, the accuracy of phase-model-based reconstruction decreases with stronger coupling, while NIPS performs consistently. We observe a similar observation in Ref. (15), where even for a small 15-node electrochemical network, 37,600 data samples are required for accurate reconstruction.

### Combining NIPS with machine learning methods

Graph neural networks have been shown to be effective for network inference (68, 69), due to their ability to learn complex dependencies in structured graph data. A typical approach involves an encoder–decoder architecture, where the encoder estimates connection probabilities based on input data and the decoder predicts future states by using these connection probabilities. Both modules are trained to minimize the error between the decoder’s predictions and the true data. However, such an approach hinges on the availability of full-state node measurements. This could be extended to partial-state observations by considering delayed observations in the learning process, as the future network state depends on the current and past state (Eq. 6). A key challenge for this extension is the design of the encoder architecture: specifically, the encoder must be constrained so that, when inferring edges involving node *i*, it only accesses the delayed states of node *i*, i.e. $\left(x_{i}(t-\tau_{i}),\ldots ,x_{i}(t-k\tau_{i})\right)$. Moreover, this strategy could be adapted to other machine learning models for network inference tasks, such as reservoir computing networks (70) and deep learning (71).

### Limitations and possible extensions

It is also important to acknowledge the assumptions and limitations of our approach. First, NIPS considers networks with fixed topology, instantaneous coupling (i.e. no coupling delay), and nodes with time-invariant dynamics. As a result, our approach is not applicable to systems where the system dynamics (node dynamics or network connectivity) are time-dependent. Extending the framework to model temporally evolving networks would require dynamic basis representations that evolve over time. Second, NIPS relies on numerical differentiation of observed time series to estimate derivatives, which makes it susceptible to measurement noise. This is, however, not the case with functional inference methods (e.g. MI and TE), which are derivative-free. In scenarios with a low signal-to-noise ratio, the functional inference methods could outperform NIPS. Future work could explore derivative-free formulations to reduce the method’s sensitivity to noise. Finally, although our method requires the measurement of all network nodes, the framework can be naturally extended to scenarios where only a subset of nodes in the network is observable. This can be achieved by viewing the network as an *Nn*-dimensional system (*N* denoting the number of nodes and *n* denoting the dimension of each node), where only *m* states are measured (*m* observable nodes with one measurement per node). The connectivity between measurable nodes can then be reconstructed using the delay embedding theorem with k≥(2Nn+1)/m, though such an extension would require substantially more data.

## Methods

### Network connectivity inference

We approximated unknown nonlinear functions $G_{i}$ and $g_{ij}$ (Eq. 6) by representing each as a linear combination of known basis functions, such as polynomials, Legendre, Fourier, or Chebyshev basis functions. Specifically, we expressed $g_{ij}(x_{j})\approx \sum_{l=1}^{r_{1}}\alpha_{l}^{\left(ij\right)}p_{l}(x_{j})$ and $G_{i}(x_{i,d})\approx \sum_{l=0}^{r}\beta_{l}^{\left(i\right)}q_{l}(x_{i,d})$, where $\left\{p_{l}\right\}$, $\left\{q_{l}\right\}$ are the known basis functions of order *l*, $\left\{\alpha_{l}^{\left(ij\right)}\right\}$, and $\left\{\beta_{l}^{\left(i\right)}\right\}$ are the coefficients to be determined, and $r_{1}$ and *r* represent the truncation order that determines the accuracy of the approximation. Each coefficient $\left\{\alpha_{l}^{\left(ij\right)}\right\}$ is scalar, while {βl(i)}∈R(k+l−1l) as $q_{l}(\cdot )$ is a multivariate function. Then, Eq. 6 can be expressed as


(9) $${\dot{x}}_{i}(t)=\sum_{l=0}^{r}\beta_{l}^{\left(i\right)}q_{l}(x_{i,d})+\sum_{\begin{array}{c} \;j=1\\ j\neq i \end{array}}^{N}\sum_{l=1}^{r_{1}}\alpha_{l}^{\left(ij\right)}p_{l}(x_{j})+\eta_{i}(t),$$

where $\eta_{i}(t)$ denotes the approximation error. Following this, the unknown coefficients $\alpha_{l}^{\left(ij\right)}$ and $\beta_{l}^{\left(i\right)}$, can be determined, given the measurement data, by solving a simple linear inverse problem


(10) $${\hat{Z}}_{i}=\arg\;\min\|Y_{i}-A_{i}Z_{i}{\|}_{2}^{2},$$

where ${\hat{Z}}_{i}=(A_{i}^{\prime }A_{i}{)}^{-1}A_{i}^{\prime }Y_{i}$ encapsulates both self-dynamics and incoming connections to node *i*; namely, Z^i=[β0(i),…,βr(i),α1i1,…,αr1i1,…,α1iN,…,αr1iN]′∈R(k+rk)+(N−1)r1. The vector $Y_{i}=[{\dot{x}}_{i}(t_{0,i}),\ldots ,{\dot{x}}_{i}(M\Delta t){]}^{\prime }$ was determined using the finite-difference method. The matrix $A_{i}=[Q_{i}\;|\;P_{1}|\ldots |P_{N}]$ is a constant matrix, where $t_{0,i}=(k-1)\tau_{i}$,


$$\begin{array}{rl} Q_{i} & =\left[\begin{array}{ccc} q_{0}(x_{i,d}(t_{0,i})) & \ldots & q_{r}(x_{i,d}(t_{0,i}))\\ q_{0}(x_{i,d}(t_{0,i}+\Delta t)) & \ldots & q_{r}(x_{i,d}(t_{0,i}+\Delta t))\\ \vdots & \ldots & \vdots \\ q_{0}(x_{i,d}(M\Delta t)) & \ldots & q_{r}(x_{i,d}(M\Delta t)) \end{array}\right],\;\text{and}\\ P_{j} & =\left[\begin{array}{ccc} \;p_{1}(x_{j}(t_{0,i})) & \ldots & p_{r_{1}}(x_{j}(t_{0,i}))\\ p_{1}(x_{j}(t_{0,i}+\Delta t)) & \ldots & p_{r_{1}}(x_{j}(t_{0,i}+\Delta t))\\ \vdots & \ldots & \vdots \\ p_{1}(x_{j}(M\Delta t)) & \ldots & p_{r_{1}}(x_{j}(M\Delta t)) \end{array}\right] \end{array}$$

for j={1,…,N}∖i. After estimating ${\hat{Z}}_{i}$, the strength of connection from node *j* to node *i*, ${\hat{k}}_{ij}$ was defined as $\sqrt{\sum_{l=1}^{r_{1}}(\alpha_{l}^{\left(ij\right)}{)}^{2}}.$ This estimation process was repeated for all the nodes, i.e. i=1,…,N.

The number of truncation terms $\left(r,r_{1}\right)$ used for the examples presented in this study were as follows: (4,1) for Rulkov maps, (3,1) for electrochemical oscillators, (7,1) for SCN networks, and (2,2) for experimental data.

### Network reconstruction accuracy evaluation

We used the AUROC metric to evaluate link prediction accuracy. The ROC curve plots the true positive rate (TPR) against the false positive rate (FPR) across various cut-off thresholds (ϵ). These thresholds convert the estimated coupling matrix ${\hat{k}}_{ij}$ into a binary matrix such that there exists a link only if ${\hat{k}}_{ij}>\epsilon$. The TPR and FPR values were calculated by comparing the estimated binary matrix with the ground truth connectivity matrix of the network. Specifically, TPR is defined as TPR = TP/(TP+FN) and FPR as FPR = FP/(FP+TN), where true positive (TP) represents the number of correctly identified connections, false negative (FN) is the number of true links missed in the inferred network, false positive (FP) is the count of falsely detected links, and true negative (TN) is the number of correctly identified negative links. A higher AUROC score indicates better classification accuracy in distinguishing true connections from false ones. An AUROC score of 0.5 indicates random guessing, whereas a score of 1.0 implies the existence of a threshold that achieves perfect network reconstruction.

## Supplementary Material

pgaf397_Supplementary_Data

## Data Availability

The data underlying this article are available in the article and in its online Supplementary material.
