# Supplementary material for: NIPS: Network Inference with Partial State measurements using forced-delay embedding
Source: PNAS Nexus. 2025 Dec 24;5(1):pgaf397. doi: 10.1093/pnasnexus/pgaf397 (PMC12770969; doi:10.1093/pnasnexus/pgaf397)
Supplement: pgaf397_Supplementary_Data [file pgaf397_supplementary_data.pdf]

Supplementary Material for

NIPS: Network Inference with Partial State Measurements Using Forced-Delay Embedding

Bharat Singhal, István Z. Kiss, and Jr-Shin Li

Jr-Shin Li
E-mail: jsli@wustl.edu

This PDF file includes:

Figs. S1 to S6
Tables S1 to S6
SI References

1. Reconstruction of neuronal connectivity structure

We consider a directed network of 20 FitzHugh-Nagumo neurons, a canonical model in computational neuroscience, to capture the activation and deactivation dynamics of spiking neurons (1). The network dynamics are governed by the dynamic law

$$\begin{aligned}\dot{V}_i &= V_i - \frac{V_i^3}{3} - W_i + I_0 + \sum_{j=1}^{20} k_{ij} a_{ij} (V_j - V_i), \\ \dot{W}_i &= aV_i - bW_i + c,\end{aligned} \qquad [1]$$

where V_i and W_i denote the membrane potential and the recovery variable of the ith neuron, respectively. The network connectivity (Supplementary Figure 1A) is determined by the binary variables a_{ij}, where $a_{ij} = 1$ indicates a link from node j to node i, and $k_{ij} \in \mathbb{R}^+$ denotes the connection strength. The model parameters a, b, c, and I_0 are selected to ensure each neuron produces a periodic spiking behavior. We simulate the network four times from random initial conditions for $T = 73$ms (2 cycles), where in each simulation, we record the membrane potential of each neuron, i.e., V_i ($i = 1, \ldots, 20$) with a rate of 500 samples per cycle. This is done to ensure that the network does not synchronize and information-rich data can be generated. We then reconstruct the network structure using data from one, two, and four simulations using NIPS, where the embedding dimension $k = 5$ as each neuron is a two-dimensional system and the embedding delay is determined using the average mutual information algorithm (2). The network reconstruction accuracy, AUROC score, increases from 0.6 to 1.0, as E increases from one to four (Supplementary Figure 1B). The final reconstructed network structure for $E = 4$ is shown in Supplementary Figure 1B, where the cut-off threshold was determined by Otsu's algorithm (3).

In addition to accurate network structure, we find that the learned network model can also be used to accurately predict the network states. To this end, we simulate the network from random initial conditions that are not used to generate the simulated data for model learning (or network inference) for 10 cycles. Then, we use the same initial conditions to evolve the learned model. The states of two randomly selected nodes are shown in Supplementary Figure 1C, where the dotted lines correspond to the predicted node states.

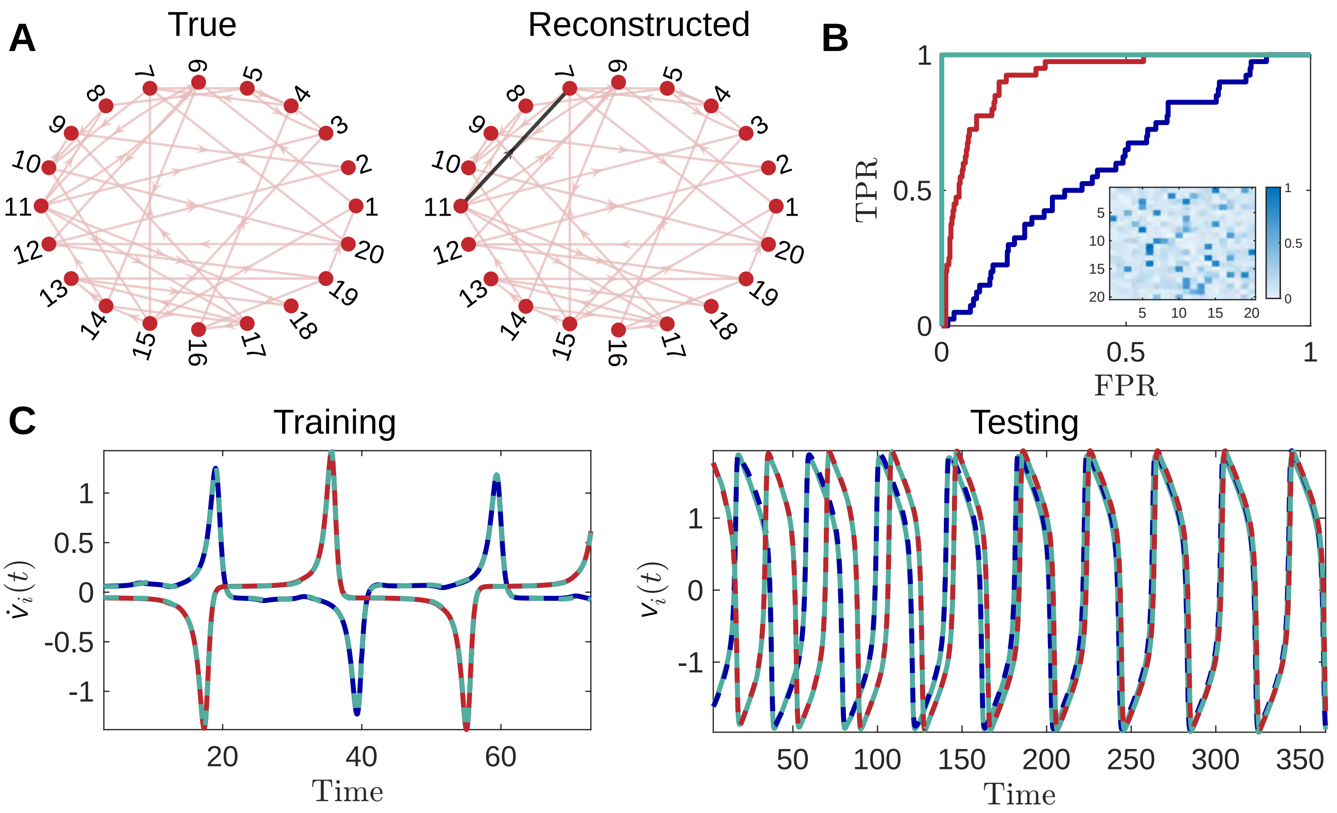

Supplementary Figure 1: Inference of a 20-node FitzHugh-Nagumo neuronal network. (A) shows the true and reconstructed network, with the black line denoting the false positive link. (B) ROC curve for three different data lengths. The blue (resp. red and green) line corresponds to one (resp. two and four) different random initial conditions used to generate data. The inset figure shows the inferred coupling matrix corresponding to the green ROC curve. The AUROC scores corresponding to the blue, red, and green lines are 0.60, 0.92, and 1.0, respectively. (C) The left panel shows the true $\dot{V}_i(t)$ (solid line) for a randomly selected simulation and fitted $\dot{V}_i(t)$ (dotted line) for two randomly selected nodes. The right panel shows the estimated trajectory of two randomly selected nodes in the network using the true model (solid) and the fitted model (dotted).

2. Connectivity inference for Rössler oscillators

We consider directed networks of 100 Rössler oscillators (sample size = 10) with 5 incoming connections per node. Each node has chaotic dynamics, given by

$$\begin{aligned}\dot{x}_i^{(1)} &= -x_i^{(2)} - x_i^{(3)} + \sum_{j=1}^{100} k_{ij} a_{ij} \sin(x_j^{(1)}) \\ \dot{x}_i^{(2)} &= x_i^{(1)} + 0.1 x_i^{(2)} \\ \dot{x}_i^{(3)} &= 0.1 + x_i^{(3)}(x_i^{(1)} - 18),\end{aligned} \quad [2]$$

where $x_i^{(1)}$, $x_i^{(2)}$ and $x_i^{(3)}$ are the states of oscillator i. We generate simulated time series data by simulating the network 50 times independently, with each simulation having 100 sample points for the state $x_i^{(1)}$. The embedding dimension k is chosen as 7, as each node is a three-dimensional system, and the delay τ_i is determined from the measurement data using the average mutual information algorithm (2). We find that even for the three-dimensional node dynamics, we can reconstruct the network accurately with only one state measurement per node (Supplementary Figure 2).

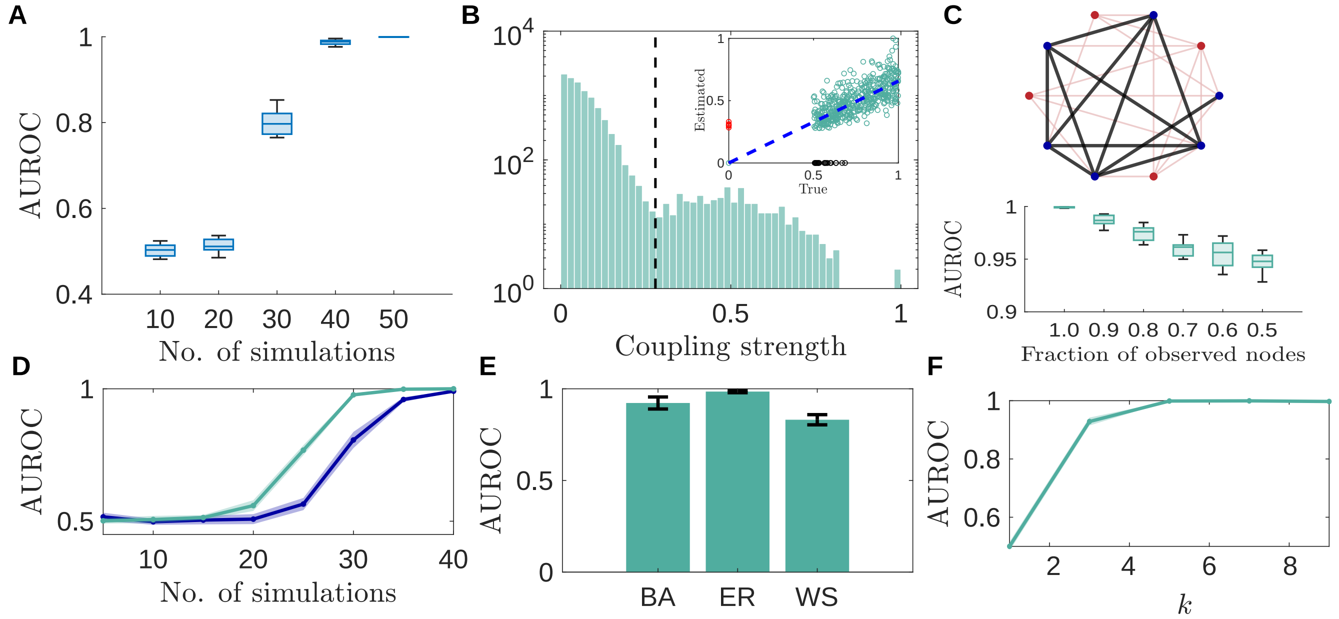

Supplementary Figure 2: Inference of 100-nodes Rössler oscillator networks. (A) AUROC score as a function of the number of independent simulations required for reconstruction. (B) Histogram of inferred coupling strengths for a representative network out of 10 networks, with the dotted black line indicating the threshold used to determine network connectivity. The inset shows the positive correlation between true and estimated coupling strengths, where red and black circles represent false positives and false negatives, respectively. (C) A sample network with observed nodes colored blue and hidden nodes red; only links between observed nodes (black lines) require recovery. The plot shows AUROC variation as different fractions of nodes are observed. (D) Comparison of AUROC scores for full (blue) and partial (green) state measurement cases, using ICON (4) for network reconstruction with complete state measurements. (E), (F) Impact of network topology and embedding dimension k on reconstruction performance, respectively.

3. Connectivity reconstruction when network dynamics are embedded

As shown in the main manuscript, if we embed the network states, then the dynamics of the observable state of node i can be expressed as

$$\dot{x}_i(t) = \boldsymbol{F}_i(\boldsymbol{x}_{1,d}, \boldsymbol{x}_{2,d}, \dots, \boldsymbol{x}_{N,d}), \qquad [3]$$

where $\boldsymbol{x}_{j,d} = (x_j(t), x_j(t-\tau), \dots, x_j(t-(k-1)\tau))' \in \mathbb{R}^k$. Now, to determine $\boldsymbol{F}_i$, we utilize the additivity assumption and decompose $\boldsymbol{F}_i$ as

$$\dot{x}_i(t) = G_i(\boldsymbol{x}_{i,d}(t)) + \sum_{j=1, j\neq i}^{N} G_{ij}(\boldsymbol{x}_{j,d}(t)). \qquad [4]$$

We then express the unknown nonlinear functions G_i and G_{ij} as a linear combination of known basis functions; namely, $G_{ij}(\boldsymbol{x}_{j,d}) \approx \sum_{l=1}^{r_1} \alpha_l^{(ij)} p_l(\boldsymbol{x}_{j,d})$ and $G_i(\boldsymbol{x}_{i,d}) \approx \sum_{l=0}^{r} \beta_l^{(i)} q_l(\boldsymbol{x}_{i,d})$, where $\{p_l\}$, $\{q_l\}$ are the known basis functions of order l, $\{\alpha_l^{(ij)}\}$ and $\{\beta_l^{(i)}\}$ are unknown coefficients that need to be determined, and r_1 and r represent the truncation order of the basis functions Note that $p_l(\cdot)$ and $q_l(\cdot)$ are multivariate functions; hence $\alpha_l^{(ij)}, \beta_l^{(i)} \in \mathbb{R}^{\binom{k+l-1}{l}}$. Equation (4), after the basis function approximation, can be expressed as

$$\dot{x}_i(t) = \sum_{l=0}^{r} \beta_l^{(i)} q_l(\boldsymbol{x}_{i,d}) + \sum_{\substack{j=1 \\ j\neq i}}^{N} \sum_{l=1}^{r_1} \alpha_l^{(ij)} p_l(\boldsymbol{x}_{j,d}) + \eta_i(t), \qquad [5]$$

where $\eta_i(t)$ denotes the approximation error. The unknown coefficients $\alpha_l^{(ij)}$ and $\beta_l^{(i)}$ are then determined by integrating the measurement data with equation (4), and solving the least-squares problem,

$$\hat{Z}_i = \arg\min \|Y_i - A_i Z_i\|_2^2, \quad [6]$$

where $\hat{Z}_i = (A_i' A_i)^{-1} A_i' Y_i$ contains both self-dynamics and incoming connections to node i; namely,

$$\hat{Z}_i = [\beta_0^{(i)}, \ldots, \beta_l^{(i)}, \alpha_1^{i1}, \ldots, \alpha_{r_1}^{i1}, \ldots, \alpha_1^{iN}, \ldots, \alpha_{r_1}^{iN}]' \in \mathbb{R}^{\binom{k+r}{k} + (N-1)(\binom{k+r_1}{k} - 1)}.$$

The vector $Y_i = [\dot{x}_i(t_0), \ldots, \dot{x}_i(M\Delta t)]'$, and the matrix $A_i = [Q_i \; |P_1 \; | \ldots \; |P_N]$, where $t_0 = (k-1)\tau$,

$$Q_i = \begin{bmatrix} q_0(\boldsymbol{x}_{i,d}(t_0)) & \ldots & q_r(\boldsymbol{x}_{i,d}(t_0)) \\ q_0(\boldsymbol{x}_{i,d}(t_0 + \Delta t)) & \ldots & q_r(\boldsymbol{x}_{i,d}(t_0 + \Delta t)) \\ \vdots & \ldots & \vdots \\ q_0(\boldsymbol{x}_{i,d}(M\Delta t)) & \ldots & q_r(\boldsymbol{x}_{i,d}(M\Delta t)) \end{bmatrix}, \text{ and}$$

$$P_j = \begin{bmatrix} p_1(\boldsymbol{x}_{j,d}(t_0)) & \ldots & p_{r_1}(\boldsymbol{x}_{j,d}(t_0)) \\ p_1(\boldsymbol{x}_{j,d}(t_0 + \Delta t)) & \ldots & p_{r_1}(\boldsymbol{x}_{j,d}(t_0 + \Delta t)) \\ \vdots & \ldots & \vdots \\ p_1(\boldsymbol{x}_{j,d}(M\Delta t)) & \ldots & p_{r_1}(\boldsymbol{x}_{j,d}(M\Delta t)) \end{bmatrix}$$

for $j = \{1, \ldots, N\} \setminus i$. The strength of connection from node j to node i, $\hat{k}_{ij}$ was defined as $\sqrt{\sum_{l=1}^{r_1} \|\alpha_l^{(ij)}\|_2^2}$. This estimation process was repeated for all the nodes, i.e., $i = 1, \ldots, N$.

4. Supplementary Tables and Figures

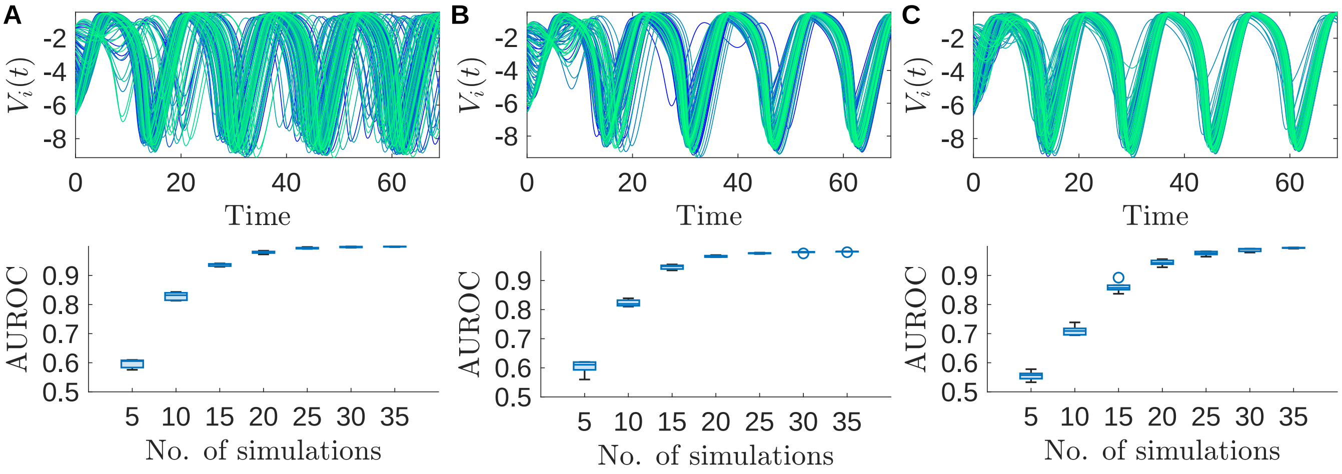

Supplementary Figure 3: NIPS accurately reconstructs networks across various synchronization regimes. The figure depicts networks of 100 directed electrochemical oscillators under three coupling strengths: weak coupling (average k_{ij}=0.01, left panel), intermediate coupling (average k_{ij}=0.02, middle panel), and strong coupling (average k_{ij}=0.03, right panel). For each panel, the top figure illustrates the time series of the oscillators for a representative simulation, while the bottom figure shows the inference accuracy (sample size: 5). Each simulation consists of five cycles with 100 samples per cycle.

5. Sensitivity and scalability analysis

A. Coupling dependence on unobserved states. In real-world systems, the coupling between nodes may partially depend on the unobserved states. To analyze the sensitivity of the NIPS algorithm to such dependencies, we consider a network of

Model Parameter	Nominal Value
Circuit Potential (U)	25
Resistance (R)	20
C_h	1600
a	0.3
Γ	0.01
b	6×10^{-5}
c	0.001
Coupling strengths (k_{ij})	$\mathcal{N}(0.01, 0.002)$

Supplementary Table 1: Model parameters for the electrochemical oscillator network, where the coupling strengths are normally distributed with mean 0.01 and standard deviation 0.002

Embedding	Number of simulations					
type	10	15	20	25	30	35
Node-wise	0.81 ± 0.04	0.93 ± 0.02	0.98 ± 0.01	0.99 ± 0.01	1.00 ± 0	1.00 ± 0
Network	0.59 ± 0.02	0.67 ± 0.03	0.77 ± 0.02	0.83 ± 0.02	0.88 ± 0.02	0.91 ± 0.01

Supplementary Table 2: AUROC mean ($\pm$ standard deviation) for 100-node electrochemical oscillators for embedding each node separately and embedding the whole network (sample size = 10). Each simulation consists of 500 sample points.

Observation	Number of simulations					
function $\varphi(X_i)$	10	15	20	25	30	35
x_i	0.81 ± 0.04	0.93 ± 0.02	0.98 ± 0.01	0.99 ± 0.01	1.00 ± 0	1.00 ± 0
x_i^2	0.6 ± 0.02	0.7 ± 0.01	0.8 ± 0.01	0.88 ± 0.01	0.93 ± 0.01	0.96 ± 0
$\frac{1}{1+e^{-x_i}}$	0.58 ± 0.02	0.71 ± 0.02	0.84 ± 0.03	0.93 ± 0.02	0.97 ± 0.01	0.99 ± 0.01

Supplementary Table 3: AUROC mean ($\pm$ standard deviation) for 100-node electrochemical oscillators for different measurement functions (sample size = 10). Each simulation consists of 500 sample points.

Model Parameter	Description	Nominal Value
K_1	mRNA transcription constant	1 nM
K_m	mRNA degradation constant	0.5 nM
$v_{m,i}$	mRNA degradation rate	[0.345,0.395] nM/h
n	mRNA transcription Hill term	4
k_s	Protein translation rate	0.417 1/h
K_d	Cytosolic protein degradation constant	0.13 nM
v_d	Cytosolic protein degradation rate	1.167 nM/h
k_1	Cytosolic to nuclear protein rate	0.417 nM/h
v_d	Nuclear to cytosolic protein rate	0.5 nM/h

Supplementary Table 4: Model parameters for the circadian oscillator networks, where v_m is uniformly distributed.

electrochemical oscillators with model parameters consistent with those used in equation (7) of the main manuscript, except for modifications in the coupling functions. We examine two distinct coupling scenarios: (i) The unobserved states ν_i are not directly coupled, but the coupling input to the observable state V_i is given by $\sum_{j=1}^{100} a_{ij} k_{ij} \left(\Delta V_{ij} + \delta \Delta \nu_{ij}\right)$. (ii) The coupling inputs to the states V_i and ν_i are $\sum_{j=1}^{100} a_{ij} k_{ij} \Delta V_{ij}$ and $\sum_{j=1}^{100} a_{ij} k_{ij} \delta \Delta \nu_{ij}$, respectively. Here, the parameter $\delta \geq 0$ quantifies the relative contribution of the unobserved states to the coupling input. The inference results are shown in Supplementary Table 5, where we collect data from 35 independent simulations, with each simulation consisting of five cycles (100 samples per cycle). We find that, under both scenarios, our method remains robust to the partial dependence of node coupling to unobserved states. We observe less than 5% degradation for moderate levels of unobserved state influence, i.e., when $\delta \leq 0.3$.

Coupling scenario	Relative contribution of the unobserved states to the coupling input δ				
	0.1	0.2	0.3	0.4	0.5
(i)	1.0 ± 0.0004	1.0 ± 0.001	1.0 ± 0.0004	1.0 ± 0.0007	1.0 ± 0.0009
(ii)	1.0 ± 0.003	0.98 ± 0.02	0.97 ± 0.02	0.83 ± 0.04	0.80 ± 0.04

Supplementary Table 5: AUROC mean ($\pm$ standard deviation) for 100-node electrochemical oscillators when coupling input depends on unobserved states (sample size = 10). In scenario (i), coupling input containing unobserved states affects only the observable state; in scenario (ii), it influences only the unobserved state.

B. Network heterogeneity. We demonstrate the generalizability of NIPS to heterogeneous node dynamics by considering a 100-node electrochemical oscillator network with increasing variability in the resistance parameter R. Specifically, R is drawn uniformly from the interval $[20, 20 - \Delta R]$, where ΔR is varied from 0 (homogeneous) to 10, corresponding to 50% heterogeneity. This variation induces differences in both oscillation type and frequency (the circuit potential $U = 20$ and all other parameters match those in Supplementary Table 1). As shown in Supplementary Figure 4, the time series of two uncoupled nodes (node 1: $R = 20$; node 85: $R = 11.5$) display markedly different dynamics. Despite this, inference results in Supplementary Table 6 indicate that NIPS maintains high accuracy across the full range of heterogeneity, demonstrating its robustness to diverse node dynamics. The number of simulations is identical to previous analysis ($E = 35$).

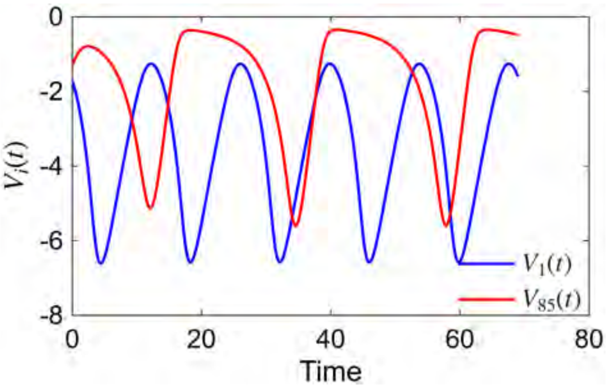

Supplementary Figure 4: Time series of two candidate nodes (node 1 and node 85) in the oscillator network, displaying heterogeneity in node dynamics.

ΔR	AUROC
0	0.98
2	1.00
4	1.00
6	1.00
8	1.00
10	1.00

Supplementary Table 6: Inference accuracy at varying heterogeneity levels (ΔR).

C. Choice of design parameters. In our implementation, we employ the monomial basis to approximate the function G_i and g_{ij} (equation (6), main manuscript). From a theoretical standpoint, under appropriate smoothness assumptions, a wide range of basis functions—such as Legendre, Chebyshev, or Fourier—can be employed to approximate these nonlinear functions. Since the choice of basis is a modeling choice, we investigate the sensitivity of the NIPS to different basis function selections. To this end, we apply NIPS to two representative network types: electrochemical oscillator ($N = 100$) and Rulkov maps (mouse visual cortex network), and reconstruct the network connectivity using two alternative basis functions—Legendre and Chebyshev polynomials of the first kind. The results (Supplementary Figure 5) demonstrate that NIPS performs consistently for all these basis function choices.

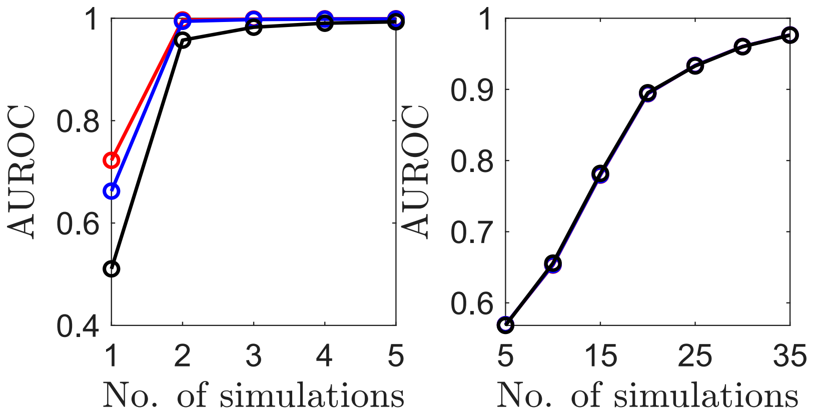

Supplementary Figure 5: Effect of choice of basis function (Legendre: blue, Chebyshev: red, and monomial: black) on the reconstruction performance for the Rulkov map (left) and electrochemical oscillator (right). The order of basis function approximation (r, r_1) are: $(5, 4)$ for the Rulkov map and $(5, 2)$ for the electrochemical oscillator.

For the Rulkov map, the orthonormal basis (Legendre and Chebyshev) slightly outperforms the monomial basis. This is because orthonormal bases lead to orthogonal (and thus linearly independent) columns in the design matrix A_i (equation (10), main manuscript), which improves numerical conditioning. However, in the large data regime, the differences in performance across basis functions diminish. In the case of the electrochemical oscillator network, the reconstruction performance is identical for all basis function choices, which is why Supplementary Figure 5 displays a single overlapping curve.

D. Scalability to large networks. We investigate how the number of required data points (or simulations) scales with network size by simulating directed networks of chaotic oscillators (equation (2)). We vary the network size N from 100 to 800 and compute the minimum number of simulations required to achieve AUROC$\geq$ 0.9, denoted as $E_{0.9}$. The average in-degree of each node increases proportionally with network size, specifically as $N/25$.

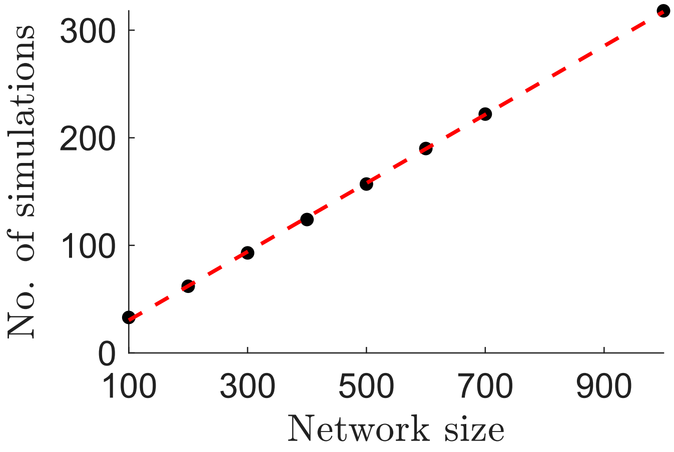

Supplementary Figure 6: Number of simulations required to achieve AUROC≥ 0.9 ($E_{0.9}$) as a function of network size.

References

1. R FitzHugh, Mathematical models of threshold phenomena in the nerve membrane. *The bulletin mathematical biophysics* **17**, 257–278 (1955).
2. B Kliková, A Raidl, Reconstruction of phase space of dynamical systems using method of time delay in *Proceedings of the 20th Annual Conference of Doctoral Students-WDS*. pp. 83–87 (2011).
3. N Otsu, , et al., A threshold selection method from gray-level histograms. *Automatica* **11**, 23–27 (1975).
4. S Wang, et al., Inferring dynamic topology for decoding spatiotemporal structures in complex heterogeneous networks. *Proc. Natl. Acad. Sci.* **115**, 9300–9305 (2018).
